# Supplementary material for: Myeloid PGGT1B Deficiency Promotes Psoriasiform Dermatitis by Promoting the Secretion of Inflammatory Factors
Source: Int J Mol Sci. 2025 May 20;26(10):4901. doi: 10.3390/ijms26104901 (PMC12112755; doi:10.3390/ijms26104901)
Supplement: Supplementary file 1 [file ijms-26-04901-s001.zip › ijms-3611085-supplementary.pdf]

Supplementary Information

Supplementary materials and methods

qRT-PCR

Total mRNA was extracted using TRIzol™ (15596018; Invitrogen, Waltham, MA, USA), following the manufacturer's instructions. Equal amounts (1 μg) of mRNA were reverse-transcribed into cDNA using the Evo M-MLV reverse transcription kit II (AG11711; Accurate Biology, Hunan, China). The cDNA served as a template and was amplified using the Roche LightCycler 480 system (Hoffmann-La Roche, Basel, Switzerland) with the SYBR® Green Premix Pro Taq HS qPCR Kit II (AG11702, Accurate Biology)

Immunohistochemistry

First, the tissue to be examined was sliced and then baked in a constant temperature box at 60°C for 20 min and soaked twice in xylene (10 min each). Then, it was soaked in anhydrous ethanol, 95% anhydrous ethanol, and 75% anhydrous ethanol for 5 minutes. Antigen repair: We placed heating sodium citrate buffer solution (DH6.0) in a microwave oven until it boiled, put the tissue chip in, cut off the power, and repeated this process 1-2 times at intervals of 5-10 min. To reduce the background interference of the experiment, the tissue samples were sealed with 5% normal goat serum and incubated at room temperature for 10 min. Incubation of primary antibody: We coated the specific rabbit primary antibody on the tissue section, put it in a wet box at 4°C overnight for incubation, took it out and returned it to room temperature, and rinsed it with PBS for 3 minutes 3 times. Incubation of secondary antibody: HRP goat anti-rabbit secondary antibody was incubated at 37°C for 30 min and washed with PBS for 3 min 3 times. Finally, horseradish peroxidase-labeled streptavidin (diluted with PBS) was added, incubated at 37°C for 30 min, and then washed with PBS for 3 min 3 times. Finally, the chromogenic reagent DAB was added for color reaction.

HE staining experiment procedure

We placed the trimmed wax block on the paraffin microtome and sliced it to a thickness of 3 μm. The slices were flattened by floating them in warm water at 42°C in a tablet-spreading machine. The tissues were picked up with slides and baked in an oven at 60 °C. We waited for the water to dry the wax, which was then removed at room temperature and saved for later. In turn, the slices were washed in xylene I, xylene II for 20 min, anhydrous ethanol I for 5 min, anhydrous ethanol II for 5 min, 95% alcohol for 5 min, 90% alcohol for 5 min, 80% alcohol for 5 min, 70% alcohol for 5 min, and distilled water for 5 min. Hematoxylin staining of nuclei: The sections were stained with hematoxylin for 5 min, washed with tap water, differentiated with 1% hydrochloric acid alcohol for several seconds, rinsed with water, turned blue with 1% ammonia water, and rinsed with running water. Eosin staining of cytoplasm: We sliced into the eosin dye solution and stained the slices for 5 min. Dehydrated sealing sheet: We placed the slices in 95% alcohol II for 15 min, anhydrous ethanol I for 10 min, anhydrous ethanol II for 10 min, xylene I for 10 min, and xylene II for 10 min to dehydrate them transparently. We then took the slices out of the xylene to dry slightly, and sealed the slices with neutral gum.

Luminex detection technology

Reagent preparation: We removed the kit from the refrigerator and balanced it at room temperature for 30 minutes. We prepared the standard products, cleaning solution, beads, antibody detection, and PE-streptavidin. Test process (according to the kit instructions): Preparation—the reagent required for the experiment was readied, and the sample was diluted well. After resuspending the beads, we added 50ul of diluted beads to each well and added 50ul of standard products and samples to each well according to the arrangement before the experiment. We kept the shaker at room temperature for 2 h, set the shaker speed to about 800rpm, and sealed the microplate. We placed the microplate on a magnetic frame for at least 1 min to ensure that the beads were adsorbed. We cleaned the beads with the cleaning solution 3 times, 100ul per hole. We added 50ul of diluted biotin-labeled detection antibody complex to each well, set the shaker speed to about 800rpm at the same room temperature for 1h, and sealed the microwell plate. We added 50ul of diluted

streptavidin-labeled PE to each well, set the shaking speed to about 800rpm at the same room temperature for 0.5 h, and sealed the microporous plate. We placed the microplate on the magnetic frame for at least 1 min to ensure that the beads were adsorbed. We cleaned the beads with cleaning solution (100ul per hole) 3 times. We re-hung the beads with 100ul cleaning solution, incubated them for 2 min, set the speed of the shaker to about 800rpm, and then tested the beads on the machine.

Bioinformatics analysis

The differential expression of mRNA was studied. We analyzed the adjusted p-value in GEO to correct false-positive results. We set the threshold as "Adjusted $p < 0.05$ and log2 (multiple change) > 1 or log2 (multiple change) < -1" to screen differentially expressed mRNA; GO was used to analyze the cellular components, molecular functions, and biological processes involved in differentially expressed genes ($p<0.05$). The related signal pathways involved in the differentially expressed genes ($p<0.05$) were analyzed using KEGG.

Western blot analysis

Cells were lysed using RIPA lysis buffer (P0013C, Beyotime) supplemented with protease and phosphatase inhibitors (Roche Applied Science, Basel, Switzerland). The protein concentrations were measured via the BCA assay (23225, Thermo Fisher Scientific, Waltham, MA, USA). Subsequently, equal amounts of denatured protein were loaded into 4–20% SmartPAGE™ Precast Protein Gel Plus (SLE020, Smart-Lifesciences, London, UK) and transferred to polyvinylidene difluoride membranes (1620177, Bio-Rad, Hercules, CA, USA). After blocking using 5% milk for 1 h, the membranes were supplemented overnight at 4 °C with primary antibodies, including PGGT1 (PA5-56529, 1:500; Invitrogen), P-p44/42 MAPK (Erk1/2) (4370S, 1:1000; Cell Signaling Technology, Danvers, MA, USA), phospho-SAPK/JNK (4668S, 1:1000; Cell Signaling Technology), P-p38 MAPK (4511S, 1:1000; Cell Signaling Technology), phospho-IκBα (2859, 1:1000; Cell Signaling Technology), IκBα (4814, 1:1000; Cell Signaling Technology), P-NF-κB p65 (3033S, 1:1000; Cell Signaling Technology), NF-κB p65 (8242, 1:1000; Cell Signaling Technology), and GAPDH (5174, 1:1000; Cell Signaling Technology). After washing, secondary antibodies were

used: anti-rabbit IgG (7074, 1:2000; Cell Signaling Technology) and anti-mouse IgG (7076, 1:2000; Cell Signaling Technology). The protein bands were visualized using the Clarity Max Western ECL Substrate (1705060, Bio-Rad).

Supplementary Figure

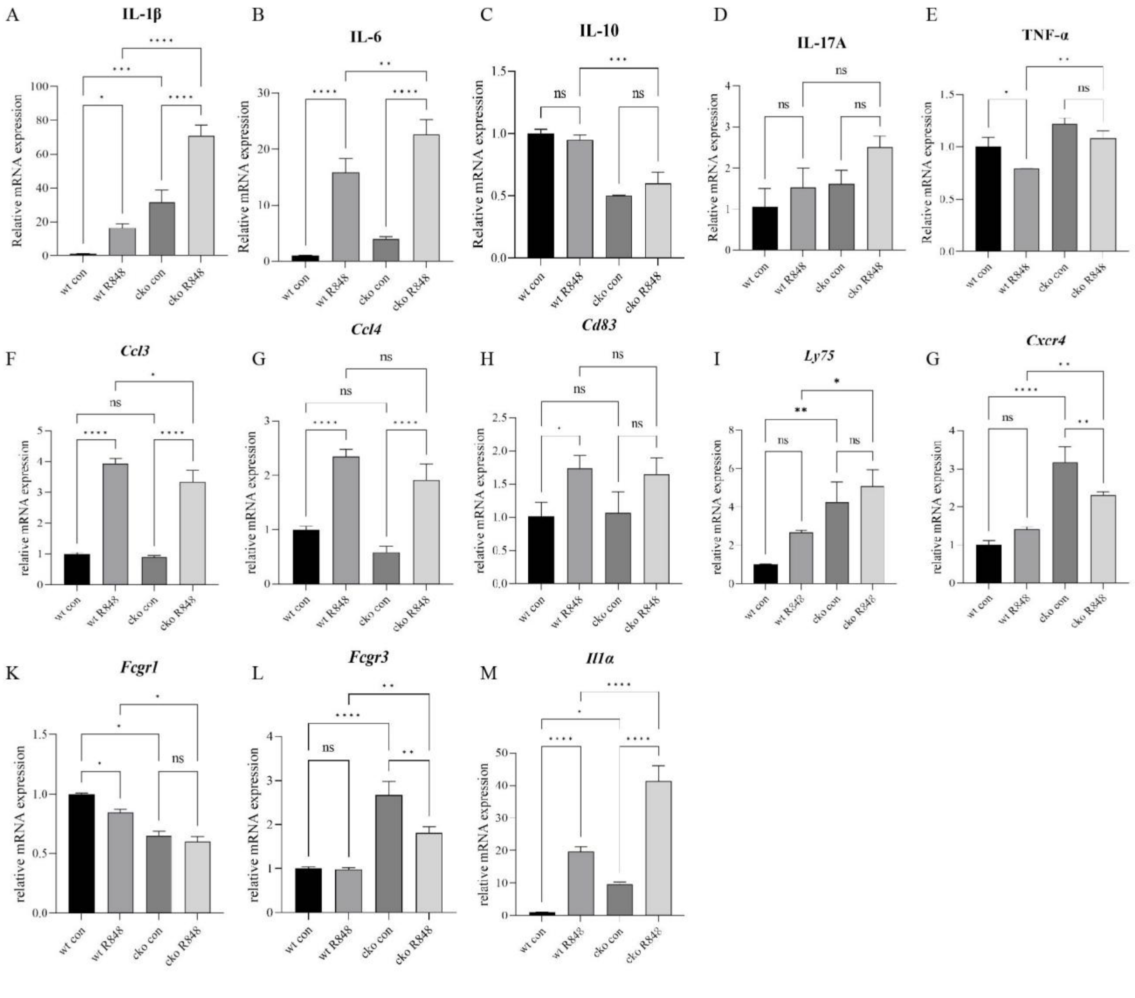

Figure S1. Pggt1b deficiency can promote the secretion of pro-inflammatory.

Quantitative real-time PCR analysis was performed to determine the mRNA levels of IL-1β, IL-6, IL-10, IL-17A, TNF-α, and the other MCODE cluster1 genes. Statistical analysis is shown. *$p < 0.05$, **$p < 0.01$, ***$p < 0.001$, and ****$p < 0.0001$. Abbreviations: wt: wild type; cko: conditional knockout; con: control; R848: resiquimod.

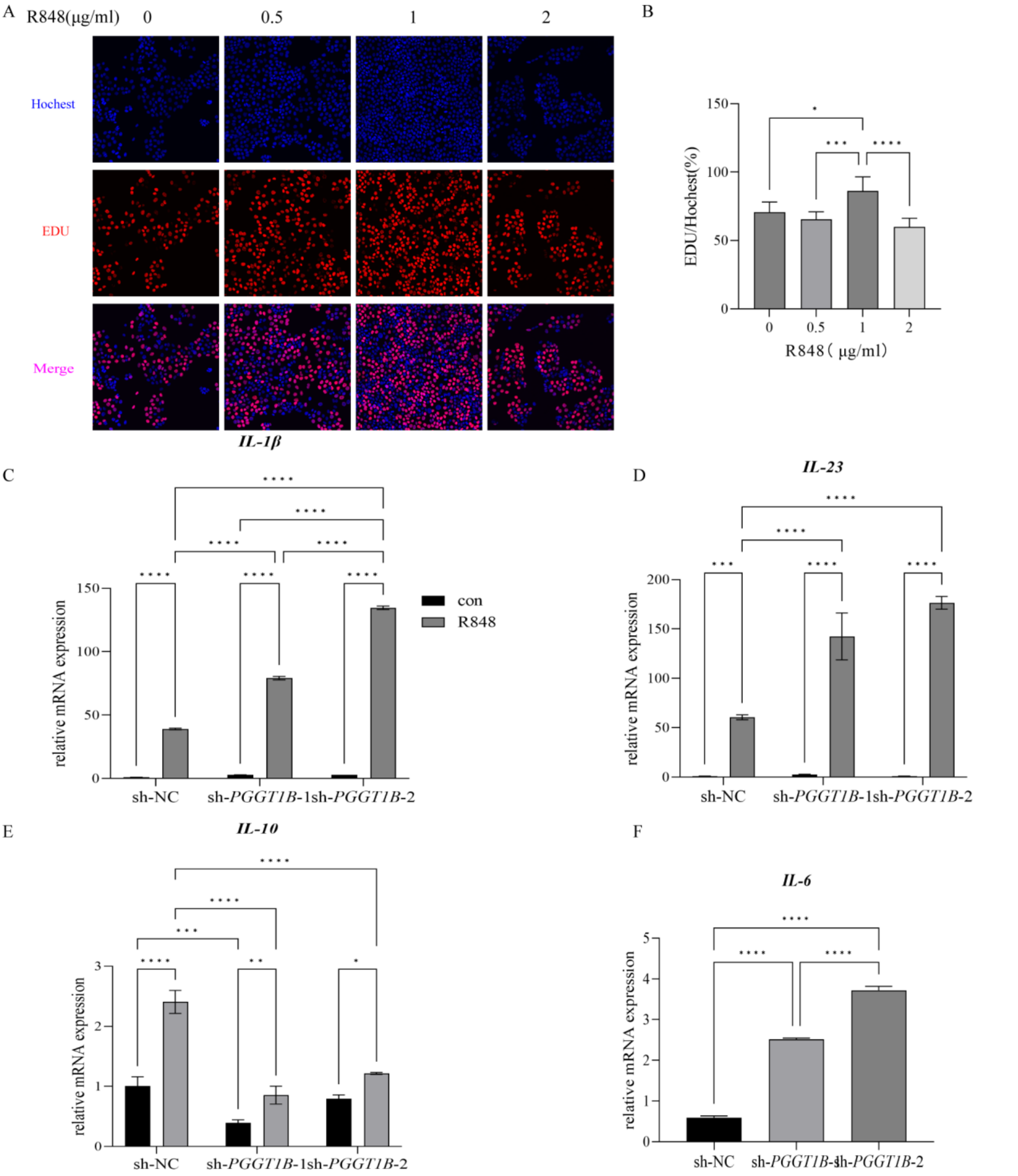

Figure S2. PGGT1B-deficient macrophages can promote the proliferation of HaCaT in the contact culture system.

(A) Effect of different concentrations of R848 on the proliferation of HaCaT. Blue: Hochest; red: EDU; 200×. (B) Statistical analysis of EdU-positive rate is shown. (C)-(F) The mRNA expression of PMA-THP-1 inflammatory factors IL-1β, IL-23, IL-10, and IL-6 were detected through qRT-PCT after R848 stimulation. Statistical analysis is shown. *$p < 0.05$, **$p < 0.01$, ***$p < 0.001$, and ****$p < 0.0001$.

Supplementary table

Supplementary Table S1. Primer sequences used in quantitative reverse transcription–polymerase chain reaction (qRT-PCR).

Gene	Forward (5‘–3’)	Reverse (5‘–3’)
Il-23	CACCTCCCTACTAGGACTCAGC	TGGGCATCTGTTGGGTCT
Cxcl1	CTGGGATTCACCTCAAGAACATC	CAGGGTCAAGGCAAGCCTC
Cxcl2	CAGACAGAAGTCATAGCCAC	TTCCAGGTCAGTTAGCCTTG
S100a7	AGCCATACTACATCACAGA	TACAGGAACTCATCAAAGC
S100a9	ACCACCATCATCGACACCTTC	AAAGGTTGCCAACTGTGCTTC
Loricrin	CTGGTGCTTCAGGGTAAC	CCAGAGGTCTTTCCACAAC
Ki67	GCCTCCTAATACACCACTGAA	GCCGTTCCTTGATGATTGTC
Serpinb2	ATCCCAAACCTGCTACCCGAA	CTCATGCGAGTTCACAGGAA
Serpinb6a	TTTGTCACCCATGAGCATATCC	CTCCATTGCCGCTGCATTTAT
Cd83	TCACATCTGGCGGAAATTGACA	GTGGCATCCCTCTGTATTGCT
Ptprc	ATATCGCGGTGTAAAACTCGT	TGGATCCCCACAACTAGGCTT
Tmtc2	AGAGAACTCAGCTCCCGTCCA	TCTGCGATTACAAAGCCGACA
lgsf6	GTCTCAAAAGCAACCGTGACC	GCTACTTATGCTCTGCCGTCT
Ly75	GCTCAGGTAATGATCCATTCACC	TTAGTTCCGCTACAGTCCTGG
Cxcl5	TCCAGCTCGCCATTCATGC	TTGCGGCTATGACTGAGGAAG
Ccl3	TTCTCTGTACCATGACACTCTGC	CGTGGAATCTTCCGGCTGTAG
Fas	TATCAAGGAGGCCCATTTTGC	TGTTTCCACTTCTAAACCATGCT
Cd274	GCTCCAAAGGACTTGTACGTG	TGATCTGAAGGGCAGCATTTC
Ccl4	TTCCTGCTGTTTCTCTTACACCT	CTGTCTGCCTCTTTTGGTCAG
Bcl2l1	TTCGGGATGGAGTAAACTGGG	AGTCATGCCCGTCCACAAAA
Fcgr1	AGGTTCCTCAATGCCAAGTGA	GCGACCTCCGAATCTGAAGA
Hif1a	ACCTTCATCGGAAACTCCAAAG	ACTGTTAGGCTCAGGTGAACT
Fcgr3	CAGAATGCACACTCTGGAAGC	GGGTCCCTTCGCACATCAG
Il1a	CGAAGACTACAGTTCTGCCATT	GACGTTTCAGAGGTTCTCAGAG
Cxcr4	GAAGTGGGGTCTGGAGACTAT	TTGCCGACTATGCCAGTCAAG
